# Validation of the Korean Version of the Clinical Frailty Scale-Adjusted Korean Triage and Acuity Scale for Older Patients in the Emergency Department

**DOI:** 10.3390/medicina60060955

**Published:** 2024-06-08

**Authors:** Ho Sub Chung, Yunhyung Choi, Ji Yeon Lim, Keon Kim, Sung Jin Bae, Yoon Hee Choi, Dong Hoon Lee

**Affiliations:** 1 Department of Emergency Medicine, Chung-Ang University Gwangmyeong Hospital, College of Medicine, Chung-Ang University, 110, Deokan-ro, Gwangmyeong-si 14353, Republic of Korea; hoshap@cau.ac.kr (H.S.C.); yunhyung0710@cau.ac.kr (Y.C.); uzimuz@cau.ac.kr (S.J.B.); 2 Department of Emergency Medicine, Ewha Womans University Seoul Hospital, College of Medicine, Ewha Womans University, 260, Gonghang-daero, Gangseo-gu, Seoul 07804, Republic of Korea; 01242s@eumc.ac.kr (J.Y.L.); mikky5163@ewha.ac.kr (K.K.); 3 Department of Emergency Medicine, Ewha Womans University Mokdong Hospital, College of Medicine, Ewha Womans University, 1071, Anyangcheon-ro, Yangcheon-gu, Seoul 07985, Republic of Korea; unii@ewha.ac.kr

**Keywords:** frailty, triage, emergency department, aged, geriatrics

## Abstract

*Background and Objectives*: The Clinical Frailty Scale (CFS), used to screen for prehospital frailty in patients aged >65 years, is simple, time-efficient, and has been validated in emergency departments (EDs). In this study, we analyzed whether the Korean Triage and Acuity Scale (KTAS) classification by level in older patients determined to have frailty based on the Korean version of the CFS increases the triage performance of the current KTAS. *Materials and Methods*: The primary outcome was 30-day in-hospital mortality, and secondary outcomes were hospital and intensive care unit (ICU) admissions. This study retrospectively analyzed prospectively collected data from three ED centers. Patients with a CFS score ranging from five (mildly frail) to nine (terminally ill) were categorized into the frailty group. We upgraded the KTAS classification of the frailty group by one level of urgency and defined this as the CFS-KTAS. *Results*: The cutoff values for predicting admission were three and two for the KTAS and CFS-KTAS, respectively. A significant difference was observed in the area under the receiver operating characteristic (AUROC) curve between the KTAS and CFS-KTAS. To predict ICU admission, the cutoff score was two for both scales. A significant difference was observed in the AUROC curve between the KTAS and CFS-KTAS. For predicting in-hospital mortality, the cutoff score was two for both scales. A significant difference was observed in the AUROC curve between the KTAS and CFS-KTAS. *Conclusions*: This study showed that the CFS-adjusted KTAS has a more useful prognostic value than the KTAS alone for predicting hospital outcomes in older patients.

## 1. Introduction

Triage in emergency departments (EDs) is a decision-making process that employs limited medical resources to identify patients requiring immediate intervention [1]. The Korean Triage and Acuity Scale (KTAS) is utilized for emergency triage, and this five-level triage tool was developed based on the Canadian Triage and Acuity Scale (CTAS) [2]. The KTAS features different classifications for adults and children (with a cutoff age of 15 years). However, it lacks consideration for older patients, resulting in the classification of healthy young adults and older patients under the same criteria. A previous study indicated poor association between the KTAS and severity in older patients (aged >65 years) compared to adults, with a higher up-triage rate observed in the older patient group [3]. Similarly, the Japan Triage and Acuity Scale, developed based on the CTAS, demonstrated lower performance in predicting hospitalization for older patients compared to adults [4]. The Manchester Triage System, another five-level system developed in the United Kingdom, also exhibited poorer performance in older patients than in younger patients [5]. Given the rapid aging of the population, a decrease in triage predictability could lead to inefficient utilization of emergency medical resources and delayed treatment for critical patients. Therefore, the implementation of different triage standards for older patients is imperative. Previous research indicated a slight improvement in triage performance when older adults were placed in a more urgent triage category [6]. Considering old age itself as a factor in triage is crucial, as older patients typically necessitate more resources than younger patients and are at a higher risk of adverse outcomes in the ED [7]. However, patients of similar age and stressors in the ED often experience different hospital outcomes depending on their underlying diseases and usual health status. Hence, identifying indicators of the personal health condition of older patients is essential, with frailty being deemed an appropriate indicator.

Frailty represents a clinical condition distinct from the typical aging process, characterized by an abnormal decline in the body’s physiological resilience to internal and external stressors, thereby increasing an individual’s vulnerability [8]. This conceptualization positions frailty not as a disease but as a clinical syndrome resulting from the disruption of multifaceted factors including genetic, biological, functional, cognitive, psychological, and socio-economic dimensions, culminating in a destabilization of homeostasis [9]. Notably, a consensus among leading international, European, and US societies advocates for frailty screening in all individuals aged over 70 [10]. The Comprehensive Geriatric Assessment (CGA) serves as the benchmark tool for identifying frailty [11], offering a multidimensional evaluation encompassing physical health, overall functional status, mental and psychosocial well-being, as well as social and environmental circumstances in geriatric populations. However, its application in busy EDs is hindered by complexity, time constraints, and the need for specialized personnel [12]. Consequently, the exigency arises for a swift and straightforward frailty screening tool tailored to the ED setting. Several validated instruments fulfill this need, including the Fatigue, Resistance, Aerobic capacity, Illnesses, and Loss of weight (FRAIL) questionnaire, Cardiovascular Health Study frailty screening scale, Gérontopôle Frailty Screening Tool, and Clinical Frailty Scale (CFS) [13,14,15,16].

The CFS, introduced by Rockwood et al., adopts a cumulative deficit approach amalgamating factors such as comorbidity, cognitive impairment, and functional limitations [17]. Comprising nine graded scales presented via visual and written aids, it classifies older adults along a spectrum from “very fit” (scale one) to “terminally ill” (scale nine). Esteemed for its simplicity and efficiency, the CFS has been validated across EDs and various European nations, serving as a prehospital frailty screening tool for individuals aged over six [18,19]. Moreover, its applicability extends to older Korean populations following translation and validation efforts [20]. Therefore, we assumed that the CFS is an appropriate tool for measuring frailty in older patients in the ED. In this study, we analyzed whether upgrading the KTAS classification of older patients determined to have frailty by one level based on the Korean version of the CFS increases the triage performance of the current KTAS.

## 2. Materials and Methods

### 2.1. Study Design and Population

This study retrospectively analyzed data prospectively collected from three ED centers in the capital of the Republic of Korea. Each of these centers is an academic tertiary hospital, collectively serving approximately 85,000 patients annually. Data were sourced from the National Emergency Department Information System (NEDIS), a registry comprising demographic and baseline clinical information from all emergency healthcare facilities overseen by the National Emergency Medical Center. Electronic medical records (EMRs) from the three hospitals were utilized. The study included all ED visits occurring between 1 August and 31 October 2023, excluding visits for non-medical issues, trauma cases, individuals dead on arrival, those discharged against medical advice, and patients under 65 years of age. Approval for this study was obtained from the Institutional Review Board of each hospital. Informed consent requirements were waived due to the retrospective and anonymized nature of the study.

### 2.2. KTAS Classification

The KTAS is a symptom-oriented classification tool that can be used by qualified doctors, nurses, and Level 1 emergency medical technicians. In this study, nurses were in charge of triage in the three EDs. Upon admission to the ED, for medical purposes, every patient was screened by a KTAS-qualified person to determine whether they had a critical first look, such as shock status, cardiac arrest, mental status change in Glasgow Coma Scale (CGS) ≤ 13 points (3–8 points are classified as KTAS Level 1, and 9–13 points are classified as KTAS Level 2), or severe difficulties in breathing. If the patients were in a life-threatening condition and required immediate medical care, they were classified as KTAS Level 1 and immediately admitted to the ED. Patients who did not meet Level 1 criteria were classified from Levels 2 to 5 based on their chief complaint and other variables [3]. The KTAS level obtained was recorded in the EMR and transmitted to NEDIS.

### 2.3. CFS Category and CFS-KTAS

Training on the CFS was provided to ED staff in the participating hospitals. The CFS category was determined for all patients aged over 65 using the Korean version of the scale. Patients with CFS scores ranging from 5 (mildly frail) to 9 (terminally ill) were categorized as frail. For this group, the KTAS level was upgraded by one level of urgency, termed the CFS-KTAS. If the initial KTAS level was 1, it remained unchanged.

### 2.4. Baseline Characteristics

Study variables, including age, sex, and vital signs at the initial ED presentation, were extracted from NEDIS and EMRs. Vital signs included systolic blood pressure (SBP), diastolic blood pressure (DBP), pulse rate (PR), respiratory rate (RR), body temperature, and mental status (assessed via the Glasgow Coma Scale). KTAS and CFS classifications were also recorded. Data on ED length of stay (LOS, minutes), disposition, in-ED mortality, and hospitalization (including ICU admission, 30-day in-hospital mortality, and hospital LOS) were collected.

### 2.5. Outcome Measures

The primary outcome was 30-day in-hospital mortality, with secondary outcomes including hospital and ICU admissions. To compare outcomes between frailty and non-frailty groups at the same KTAS level, hospital outcomes, admission rates, ICU admission rates, 30-day in-hospital mortality, and hospital LOS were assessed for each KTAS category.

### 2.6. Statistical Analysis

Continuous variables are expressed as mean ± standard deviation (SD) or median (interquartile range), and categorical variables are expressed as numbers and percentages. The independent *t*-test or Mann–Whitney U test was used for continuous variables. Pearson’s chi-square or Fisher’s exact tests were used for nominal variables. Univariate and multivariate logistic regression analyses were used to predict primary and secondary outcomes. The odds ratios (ORs) and 95% confidence intervals (CIs) were reported. The optimal cutoff value, sensitivity, specificity, and area under the receiver operating characteristic (AUROC) curve were analyzed to compare the adequacy of the KTAS and CFS-KTAS. The optimal cutoff value was defined as the point at which the value of “sensitivity + specificity − 1” was at its maximum (Youden’s index) [21]. AUROC levels between 0.8 and 0.9, 0.7 and 0.8, and 0.6 and 0.7 indicated good, suitable, and low predictive capability, respectively [22]. Statistical analyses were performed using IBM SPSS Statistics (version 26.0; IBM Corporation, Armonk, NY, USA), and the AUROC curves were analyzed using the DeLong method using MedCalc statistical software (version 19; MedCalc Software Bvba, Ostend, Belgium). In this study, *p* < 0.05 was considered statistically significant.

## 3. Results

### 3.1. General and Clinical Characteristics

**Figure 1:**
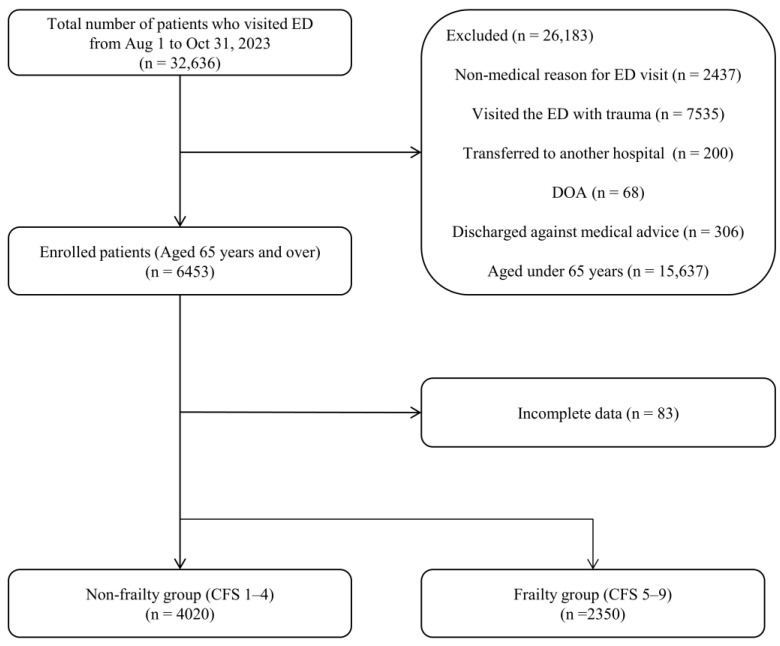
Flow chart of participant selection. ED: emergency department; CFS: Clinical Frailty Scale; and DOA: dead on arrival.

A total of 32,636 patients visited the ED between 1 August and 31 October 2023. Among them, 6453 were enrolled patients. We excluded 83 patients with incomplete data; the non-frailty group comprised 4020 patients, and the frailty group comprised 2350 patients (Figure 1). The general clinical characteristics are presented in Table 1. The mean age was 74.6 ± 7.12 years in the non-frailty group and 80.3 ± 7.97 years in the frailty group (*p* < 0.001). The proportion of females was higher in the frailty group than in the non-frailty group (53.2% [2141] vs. 56.4% [1327], *p* = 0.013). Compared to the non-frailty group, the frailty group exhibited significantly lower SBP (141.6 ± 28.18 vs. 133.4 ± 31.13 mmHg, *p* < 0.001) and DBP (76.2 ± 19.71 vs. 70.8 ± 17.25 mmHg, *p* < 0.001) and a higher PR (85.1 ± 19.71 vs. 89.1 ± 22.10 beats/min, *p* < 0.001) and RR (19.8 ± 1.90 vs. 20.2 ± 3.06 cycles/min, *p* < 0.001).

The proportion of patients exhibiting altered mental status was significantly higher in the frailty group compared to the non-frailty group (1.8% (74) vs. 6.5% (155), *p* < 0.001). There were more patients classified as urgent KTAS Levels 1 and 2 in the frailty group than in the non-frailty group, with a notable disparity between the two groups (*p* < 0.001). Significant differences were noted between the two groups in terms of ED LOS (182 (119–266) min in the non-frailty group and 233 (154–338) min in the frailty group) (*p* < 0.001) and discharge rate (59.4% (2390) in the non-frailty group and 40.1% (944) in the frailty group) (*p* < 0.001). In-ED mortality (0.02% (1) vs. 0.7% (17)) and admission rates (40.5% (1629) vs. 59.1% (1389)) were higher in the frailty group than in the non-frailty group, with statistically significant differences (*p* < 0.001). Upon admission, the frailty group demonstrated a higher proportion of patients admitted to the ICU (9.2% (373) vs. 17.1% (402)) and a 30-day in-hospital mortality rate (72 [1.7%] vs. 154 [9%]) compared to the non-frailty group (*p* < 0.001). Hospital LOS was significantly lengthier in the frailty group compared to the non-frailty group (8 [5.00–14.00] vs. 10 [6.00–17.00] days, *p* < 0.001).

### 3.2. Comparison of Patient Prognosis by Frailty Status in Each KTAS Category

The admission rate, ICU admission rate, 30-day in-hospital mortality rate, and hospital LOS were compared for each KTAS level in both groups (Table 2). In KTAS Level 1, there were 15 and 25 patients included in the non-frailty and frailty groups, respectively. No statistically significant differences were observed in the admission rate (100% (15) vs. 84% (21), *p* = 0.102) or 30-day in-hospital mortality (66.7% (10) vs. 56% (14), *p* = 0.505). The ICU admission rate was statistically significantly higher in the non-frailty group than in the frailty group (100% (15) vs. 72% (18), *p* = 0.033). Hospital LOS was longer in the frailty group than in the non-frailty group; however, no statistically significant differences were observed (7 [4.00–11.00] vs. 9 [2.00–20.50] days, *p* = 0.619). In KTAS Level 2, there were 497 and 516 patients included in the non-frailty and frailty groups, respectively. The admission rate (73% (363) vs. 83.9% (433), *p* < 0.001) and 30-day in-hospital mortality (7.6% (38) vs. 16.4% (85), *p* < 0.001) were statistically significantly higher in the frailty group than in the non-frailty group. No statistically significant differences were observed in the ICU admission rate (38.6% (192) vs. 42% (219), *p* = 0.267) and hospital LOS (9 [5.00–19.00] vs. 11 [6.00–19.00] days, *p* = 0.280). In KTAS Level 3, statistically significant differences were observed between the two groups in the admission rate (12.7% (64) vs. 18.0% (38), *p* < 0.001), ICU admission rate (5.4% (161) vs. 10.4% (166), *p* < 0.001), 30-day in-hospital mortality (0.8% (24) vs. 3.2% (52), *p* < 0.001), and hospital LOS (7 [5.00–12.00] vs. 9 [5.00–16.00] days, *p* < 0.001). In KTAS Level 4, 30-day in-hospital mortality (0% (0) vs. 1.4% (3), *p* < 0.001) was statistically significantly higher in the frailty group than in the non-frailty group. In KTAS Level 5, the admission (1.6% (1) vs. 7.6% (1), *p* < 0.001) was statistically significantly higher in the frailty group than in the non-frailty group.

### 3.3. Univariate and Multivariate Logistic Regression Analyses for Predicting Outcomes

Univariate regression analysis (Table 3) revealed significant associations between age, sex, CFS, GCS, KTAS, and CFS-KTAS with patient outcomes, such as 30-day in-hospital mortality, ICU admission, and overall admission. In the multivariate regression analysis, age, sex, CFS, GCS, KTAS, and CFS-KTAS were identified as significant predictors of mortality. Moreover, GCS, KTAS, and CFS-KTAS emerged as significant predictors for ICU admission, while sex, GCS, KTAS, and CFS-KTAS were significant predictors for overall admission.

### 3.4. Cutoff Value, AUROC Curve, Sensitivity, and Specificity for Predicting Prognosis Using the KTAS and CFS-KTAS

**Figure 2:**
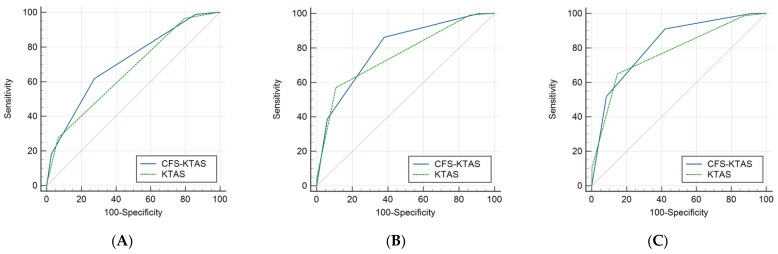
Comparison of the AUROC curves between the KTAS and CFS-KTAS. (**A**) For predicting admission; (**B**) for predicting ICU admission; and (**C**) for predicting 30-day in-hospital mortality. AUROC: area under the receiver operating characteristic; KTAS: Korean Triage and Acuity Scale; CFS: Clinical Frailty Scale; and ICU: intensive care unit.

The cutoff values, AUROC, sensitivity, and specificity for predicting admission, ICU admission, and 30-day in-hospital mortality are presented in Table 4, with a comparison of the AUROC curves illustrated in Figure 2. The cutoff value for predicting admission was three for the KTAS and two for the CFS-KTAS. A significant difference was noted in the AUROC curves between the KTAS (0.633) and CFS-KTAS (0.711) (*p* < 0.001). For predicting ICU admission, the cutoff score was two for both scales, with a statistically significant difference observed in the AUROC curves between the KTAS (0.759) and CFS-KTAS (0.795) (*p* < 0.001). In predicting in-hospital mortality, the cutoff was two for both scales, with a significant difference observed in the AUROC curves between the KTAS (0.775) and CFS-KTAS (0.819) (*p* < 0.001).

## 4. Discussion

In this study, we found that the CFS-KTAS was better than the conventional KTAS in predicting admission, ICU admission, and 30-day in-hospital mortality in older patients. As the conventional KTAS is poorly associated with severity in older patients, this result suggests that the CFS can be used to adjust the current system and determine how it should develop or change in this aging era. Older patients may have multiple chronic diseases, cognitive impairment, and atypical presentations of common diseases, making it more challenging to triage them accurately [23]. These characteristics may lead to underestimating the triage of older patients in the ED, leading to delayed care and potential morbidity [24]. The CFS can correct this underestimation in the current triage.

Several previous studies have predicted CFS-related outcomes in the ED. The CFS may be associated with a poor prognosis and extended hospital LOS in patients with sepsis in the ED [25]. A high CFS score during ED triage is associated with adverse outcomes in older adults [26]. Three frailty scales, including the CFS, are useful for identifying frail older patients at a high risk of developing adverse outcomes [27]. However, little research has been conducted on the application of frailty in triage. Ng et al. published a study on the application of the CFS to the Taiwan Triage and Acuity Scale (TTAS) in 2023. They also showed that upgrading older patients by one triage level, from a CFS level of four to nine, had greater predictive power for ICU admission and in-hospital mortality, similar to our findings [28]. Comparable results were observed for the two triage systems, KTAS and TTAS (both developed based on the CTAS), suggesting that frailty screening (an easy and quick form of the CFS) may be considered and adjusted during the triage process of older patients. Therefore, follow-up studies are required in areas that use other triage systems. If comparable outcomes emerge, the CFS could be considered and applied to advance the triage systems.

Elevating the triage level of all frail patients in congested ED settings can significantly stress medical staff. According to the KTAS guidelines, Level 1 patients must receive immediate treatment, without any delay. Levels 2 and 3 patients should undergo re-evaluation every 10 and 30 min, respectively [3]. Consequently, if a considerable number of patients require simultaneous treatment, ED overcrowding may exacerbate, leading to prolonged wait times for critically ill patients and heightened rates of unnecessary admissions [29,30]. Thus, implementing the methodology proposed in this study in practical settings necessitates thorough deliberation and discourse.

Another critical aspect in the triage of elderly patients revolves around the decision making concerning life-sustaining treatment (LST). In end-of-life scenarios (such as sudden death, organ failure, or terminal illness), patients or their families may need to determine treatment objectives and whether to opt for aggressive medical interventions [23]. Consequently, instances are often observed where patients with high urgency levels may decline immediate or aggressive medical interventions. This concern was also noted in the current study. Regarding KTAS Level 2, the frailty group exhibited higher admission rates and 30-day in-hospital mortality rates. For KTAS Level 3, the frailty group displayed higher admission rates, ICU admission rates, 30-day in-hospital mortality rates, and longer hospital LOS. In KTAS Level 4, the frailty group experienced higher 30-day in-hospital mortality rates compared to the non-frailty group. However, for KTAS Level 1, the ICU admission rate was lower in the frailty group than in the non-frailty group. This observation suggests that guardians or patients might opt out of further futile LST and choose to forgo aggressive treatments. It was corroborated that these contradictory results were exemplified by a patient who suffered cardiac arrest during their ED stay but declined cardiopulmonary resuscitation and was discharged from the ED due to a refusal of aggressive treatment.

Ideally, decisions concerning the appropriateness of LST should be made prior to an ED visit. However, in South Korea, many older patients presenting in critical condition to the ED have not yet made decisions regarding LST. Frequently, they encounter information about LST for the first time during their ED visit, leading to hurried decisions made without adequate explanation and understanding, occasionally disregarding the patient’s preferences. In contrast to the United States, where the “Natural Death Act” was established in 1976 to provide guidelines for discontinuing futile LST, South Korea saw the enactment of the “Act on Hospice and Palliative Care and Decisions on Life-Sustaining Treatment for Patients at the End-of-Life” on 3 February 2016, which took effect on 4 February 2018 [31]. Post-implementation studies revealed that only 973.8 out of every 100,000 individuals aged ≥ 19 years had completed advance directives for LST, compared to one in three adults in the United States [32]. Therefore, there is a pressing need for increased public awareness, thorough discussions, and informed decisions involving patients, medical personnel, and families. Encouraging patients to make LST decisions beforehand with a clear understanding of treatment objectives could alleviate triage fatigue in the ED.

Recently, many studies that use frailty to predict patient outcomes in ED have been published. The increased aging population is a global trend, and it may reflect the growing interest in this topic as demand for ED services is expected to rise among geriatric patients. This study also documented that the frailty group had longer ED and hospital LOS and higher mortality and admission rates. Moreover, even at the same KTAS level, the admission and mortality rates of the frailty group were high. Similar to this study, the CFS assessed at the ED is associated with adverse outcomes and mortality in older patients [26,33]. Evaluating frailty in the ED is essential because the outcomes of frailty patients are poor. Among the frailty screening tools, the CFS is one of the most used and is suitable for use in the ED [34,35]. We found in this study that the CFS is helpful for frailty assessment in older patients who visited the ED and can be applied to the KTAS to help predict patient outcomes.

### Limitations

This study has some limitations. First, there is no gold standard method for triage accuracy. The triage scale was designed to evaluate the patient’s urgency. However, several previous studies selected admission, ICU admission, and mortality rates as outcomes of triage performance [3,4,5,6,28]. Moreover, if patients were admitted or died, this could reflect the urgency at the triage level. Thus, we believe that the admission, ICU admission, and in-hospital mortality rates can represent triage outcomes. Second, selection bias may have been introduced because this study was conducted in South Korea; most of the participants were Asian and all were patients who had visited the ED of a university hospital located in the capital area. Although we attempted to minimize this bias by expanding the study to three centers, follow-up studies in various regions using different triage systems are required. Third, as ED doctors and nurses concurrently judged the CFS and clinical decisions, a confirmatory bias may have been in the results.

## 5. Conclusions

This study showed that the CFS-adjusted KTAS has a more useful prognostic value than the KTAS alone for predicting hospital outcomes in older patients. By carefully considering other factors applicable to older patients and further research conducted in various regions and on a larger scale, it would be helpful to apply the CFS in the triage of older patients whose urgency levels could be easily underestimated.

## Figures and Tables

**Table 1:** General and clinical patient characteristics.

Variable	Non-Frailty Group (CFS 1–4)	Frailty Group (CFS 5–9)	
	*n* = 4020	*n* = 2350	*p*-Value
Age (years) ^a^	74.6 ± 7.12	80.3 ± 7.97	<0.001
Sex ^b^			0.013
Male	1879 (46.7)	1023 (43.5)	
Female	2141 (53.2)	1327 (56.4)	
Vital signs ^a^			
Systolic blood pressure (mmHg)	141.6 ± 28.18	133.4 ± 31.31	<0.001
Diastolic blood pressure (mmHg)	76.2 ± 19.71	70.8 ± 17.25	<0.001
Pulse rate (beats/min)	85.1 ± 19.71	89.1 ± 22.10	<0.001
Respiratory rate (breath/min)	19.8 ± 1.90	20.2 ± 3.06	<0.001
Body temperature (°C)	36.9 ± 1.04	36.8 ± 2.39	0.204
Altered mental status ^b^	74 (1.8)	155 (6.5)	<0.001
KTAS triage category ^b^			<0.001
Level 1 Resuscitation	15 (0.3)	25 (1.0)	
Level 2 Emergent	497 (12.3)	516 (21.9)	
Level 3 Urgent	2946 (73.2)	1585 (67.4)	
Level 4 Less urgent	502 (12.4)	211 (8.9)	
Level 5 Non-urgent	60 (1.4)	13 (0.5)	
ED LOS (min) ^c^	182 (119–266)	233 (154–338)	<0.001
ED disposition ^b^			<0.001
Discharge ^b^	2390 (59.4)	944 (40.1)	
Admission ^b^	1629 (40.5)	1389 (59.1)	
In-ED mortality ^b^	1 (0.02)	17 (0.7)	
ICU admission ^b^	373 (9.2)	402 (17.1)	<0.001
30-day in-hospital mortality ^b^	72 (1.7)	154 (6.5)	<0.001
Hospital LOS (day) ^c^	8 (5.00–14.00)	10 (6.00–17.00)	<0.001

^a^ Data are presented as mean ± standard deviation. ^b^ Data are presented as numbers (%). ^c^ Data are presented as median (interquartile range). CFS: Clinical Frailty Scale; KTAS: Korean Triage and Acuity Scale; ED: emergency department; LOS: length of stay; and ICU: intensive care unit.

**Table 2:** Comparison of patient prognosis by frailty status in each KTAS category.

Variable	Non-Frailty Group (CFS 1–4)	Frailty Group (CFS 5–9)	
	*n* = 4020	*n* = 2350	*p*-Value
KTAS triage Level 1	*n* = 15	*n* = 25	
Admission ^a^	15 (100)	21 (84.0)	0.102
ICU admission ^a^	15 (100)	18 (72.0)	0.033
30-day in-hospital mortality ^a^	10 (66.7)	14 (66.7)	0.505
Hospital LOS ^b^	7 (4.00–11.00)	9 (2.00–20.50)	0.619
KTAS triage Level 2	*n* = 497	*n* = 516	
Admission ^a^	363 (73.0)	433 (83.9)	<0.001
ICU admission ^a^	192 (38.6)	217 (42.0)	0.267
30-day in-hospital mortality ^a^	38 (7.6)	85 (16.4)	<0.001
Hospital LOS ^b^	9 (5.00–19.00)	11 (6.00–19.00)	0.280
KTAS triage Level 3	*n* = 2946	*n* = 1585	
Admission ^a^	1186 (40.2)	896 (59.5)	<0.001
ICU admission ^a^	161 (5.4)	166 (10.4)	<0.001
30-day in-hospital mortality ^a^	24 (0.8)	52 (3.2)	<0.001
Hospital LOS ^b^	7 (5.00–12.00)	9 (5.00–16.00)	<0.001
KTAS triage Level 4	*n* = 502	*n* = 211	
Admission ^a^	64 (12.7)	38 (18.0)	0.067
ICU admission ^a^	5 (0.9)	1 (0.4)	0.486
30-day in-hospital mortality ^a^	0 (0.0)	3 (1.4)	0.007
Hospital LOS ^b^	8 (5.00–10.00)	9 (5.75–12.00)	0.603
KTAS triage Level 5	*n* = 60	*n* = 13	
Admission ^a^	1 (1.6)	1 (7.6)	<0.001
ICU admission ^a^	0 (0.0)	0 (0.0)	
30-day in-hospital mortality ^a^	0 (0.0)	0 (0.0)	
Hospital LOS ^b^	2	5	

^a^ Data are presented as numbers (%). ^b^ Data are presented as median (interquartile range). CFS: Clinical Frailty Scale; KTAS: Korean Triage and Acuity Scale; LOS: length of stay; and ICU: intensive care unit.

**Table 3:** Logistic regression analysis of outcome predictors.

Variable	Univariate Analysis			Multivariate Analysis		
	Odds Ratio	95% CI	*p*-Value	Odds Ratio	95% CI	*p*-Value
Predicting 30-day in-hospital mortality						
Age (65–85 vs. ≥85 years)	2.375	1.790–3.151	<0.001	1.659	1.210–2.274	<0.001
Sex (Male vs. Female)	0.731	0.560–0.954	0.021	0.723	0.560–0.954	0.028
CFS (1–3 vs. 4–9)	3.845	2.893–5.112	<0.001	1.656	1.139–2.408	0.008
GCS (3–13 vs. 13–15)	19.319	14.032–26.599	<0.001	5.003	3.43–7.229	<0.001
KTAS (1–2 vs. 3–5)	10.758	8.111–14.268	<0.001	3.745	2.532–5.539	<0.001
CFS-KTAS (1–2 vs. 3–5)	11.196	7.466–16.790	<0.001	2.573	1.448–4.572	0.001
Predicting ICU admission						
Age (65–85 vs. ≥85 years)	1.410	1.178–1.689	<0.001			
Sex (Male vs. Female)	0.781	0.672–0.908	0.001			
CFS (1–3 vs. 4–9)	2.081	1.734–2.347	<0.001			
GCS (3–13 vs. 13–15)	14.202	10.742–18.777	<0.001	3.165	2.338–4.338	<0.001
KTAS (1–2 vs. 3–5)	10.827	9.179–12.772	<0.001	4.915	3.976–6.077	<0.001
CFS-KTAS (1–2 vs. 3–5)	6.394	5.342–7.652	<0.001	2.470	1.975–3.088	<0.001
Prediction admission						
Age (65–85 vs. ≥85 years)	1.441	1.269–1.637	<0.001			
Sex (Male vs. Female)	0.724	0.656–0.799	<0.001	0.723	0.655–0.809	<0.001
CFS (1–3 vs. 4–9)	2.121	1.913–2.353	<0.001			
GCS (3–13 vs. 13–15)	8.132	5.491–12.044	<0.001	2.350	1.546–3.571	<0.001
KTAS (1–2 vs. 3–5)	5.392	4.604–6.316	<0.001	2.454	2.034–2.959	<0.001
CFS-KTAS (1–2 vs. 3–5)	3.595	3.237–3.992	<0.001	2.491	2.207–2.810	<0.001

CI: Confidence interval; CFS: Clinical Frailty Scale; GCS: Glasgow Coma Scale; KTAS: Korean Triage and Acuity Scale; and ICU: intensive care unit.

**Table 4:** KTAS and CFS-KTAS cutoff values, AUROC curve, sensitivity, and specificity for predicting prognosis.

	Cutoff Value	AUROC Curve (95% CI)	Sensitivity, % (95% CI)	Specificity, % (95% CI)
KTAS score				
For predicting admission	3	0.673	27.3 (25.6–29.7)	94.8 (92.0–95.3)
For predicting ICU admission	2	0.793	92.9 (55.6–59.5)	57.1 (88.5–91.2)
For predicting 30-day in-hospital mortality	2	0.806	90.5 (62.5–66.8)	61.1 (83.8–87.5)
CFS-KTAS score				
For predicting admission	2	0.704	89.2 (25.6–29.7)	39.2 (92.0–95.3)
For predicting ICU admission	2	0.813	83.9 (55.6–59.5)	71.1 (88.5–91.2)
For predicting 30-day in-hospital mortality	2	0.853	81.4 (62.5–66.8)	88.1 (83.8–87.5)

AUROC: area under the receiver operating characteristic; ICU: intensive care unit; KTAS: Korean Triage and Acuity Scale; CFS: Clinical Frailty Scale; and CI: confidence interval.

## Data Availability

The data that support the findings of this study are available from the first author (H.S.C.) upon reasonable request.
